# 1-[2-Oxo-1′-phenyl-2′,3′,5′,6′,7′,7a’-hexa­hydroindoline-3-spiro-3′-1′*H*-pyrrolizin-2′-yl]-3-phenyl­prop-2-en-1-one

**DOI:** 10.1107/S1600536808024781

**Published:** 2008-08-23

**Authors:** S. Nirmala, R. Murugan, E. Theboral Sugi Kamala, L. Sudha, S. Sriman Narayanan

**Affiliations:** a Department of Physics, Easwari Engineering College, Ramapuram, Chennai 600 089, India; b Department of Analytical Chemistry, University of Madras, Guindy Campus, Chennai 600 025, India; c Department of Physics, SRM University, Ramapuram Campus, Chennai 600 089, India

## Abstract

In the title compound, C_29_H_26_N_2_O_2_, one of the pyrrolidine rings in the pyrrolizine system is disordered, with site occupancies of *ca* 0.55 and 0.45. Both components of the disordered pyrrolidine ring adopt envelope conformations, whereas the other pyrrolidine ring adopts a twist conformation. The mol­ecules are linked into centrosymmetric dimers by N—H⋯O hydrogen bonds and the dimers are connected *via* C—H⋯π inter­actions.

## Related literature

For related literature, see: Araki *et al.* (2002 ▶); Caine (1993 ▶); Gore *et al.* (1991 ▶); Harris & Uhle (1960 ▶); Ho *et al.* (1986 ▶); James *et al.* (1991 ▶); Kobayashi *et al.* (1991 ▶); Ramesh *et al.* (2007 ▶); Stevenson *et al.* (2000 ▶); Tietze *et al.* (1988 ▶). For ring puckering parameters, see: Cremer & Pople (1975 ▶).
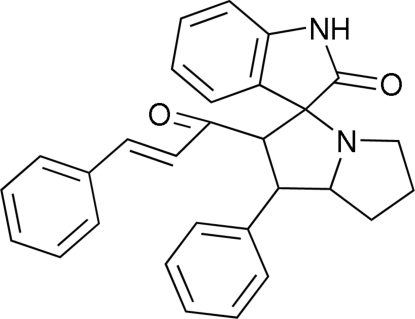


## Experimental

### 

#### Crystal data

C_29_H_26_N_2_O_2_
*M* *_r_* = 434.52
Triclinic, 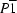

*a* = 8.4210 (2) Å
*b* = 11.8895 (3) Å
*c* = 12.5121 (3) Å
α = 95.662 (1)°
β = 105.071 (1)°
γ = 105.815 (1)°
*V* = 1144.31 (5) Å^3^
*Z* = 2
Mo *K*α radiation
μ = 0.08 mm^−1^
*T* = 293 (2) K
0.30 × 0.20 × 0.16 mm

#### Data collection

Bruker Kappa APEXII diffractometer
Absorption correction: multi-scan (Blessing, 1995 ▶) *T*_min_ = 0.977, *T*_max_ = 0.987
30483 measured reflections
7422 independent reflections
4682 reflections with *I* > 2σ(*I*)
*R*_int_ = 0.024

#### Refinement

*R*[*F* ^2^ > 2σ(*F* ^2^)] = 0.058
*wR*(*F* ^2^) = 0.200
*S* = 1.04
7422 reflections
308 parameters
H-atom parameters constrained
Δρ_max_ = 0.43 e Å^−3^
Δρ_min_ = −0.23 e Å^−3^

### 

Data collection: *APEX2* (Bruker, 2004 ▶); cell refinement: *APEX2* and *SAINT* (Bruker, 2004 ▶); data reduction: *SAINT* and *XPREP* (Bruker, 2004 ▶); program(s) used to solve structure: *SHELXS97* (Sheldrick, 2008 ▶); program(s) used to refine structure: *SHELXL97* (Sheldrick, 2008 ▶); molecular graphics: *ORTEP-3* (Farrugia, 1997 ▶); software used to prepare material for publication: *PLATON* (Spek, 2003 ▶).

## Supplementary Material

Crystal structure: contains datablocks I, global. DOI: 10.1107/S1600536808024781/ci2643sup1.cif

Structure factors: contains datablocks I. DOI: 10.1107/S1600536808024781/ci2643Isup2.hkl

Additional supplementary materials:  crystallographic information; 3D view; checkCIF report

## Figures and Tables

**Table 1:** Hydrogen-bond geometry (Å, °)

*D*—H⋯*A*	*D*—H	H⋯*A*	*D*⋯*A*	*D*—H⋯*A*
N2—H2⋯O2^i^	0.86	2.02	2.854 (2)	162
C28—H28⋯*Cg*1^ii^	0.93	2.89	3.815 (3)	172

Symmetry codes: (i) 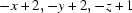; (ii) 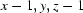. *Cg*1 is the centroid of the C8–C13 ring.

**Fig. 1.**
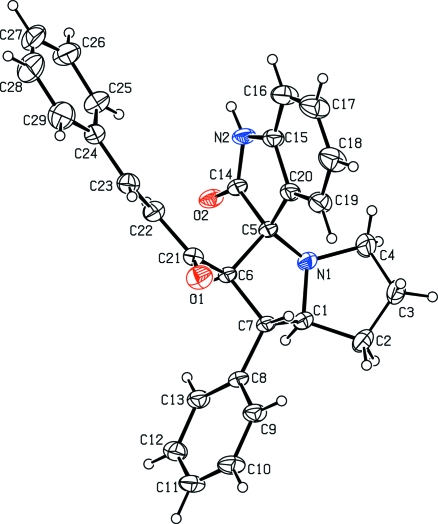
The molecular structure of the title compound with 30% probability displacement ellipsoids. Only the major disorder component is shown.

**Fig. 2.**
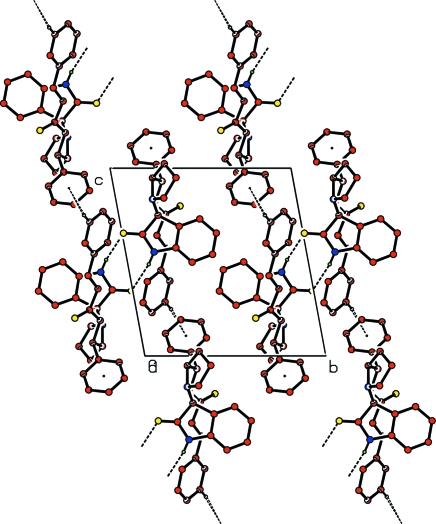
The packing of the molecules viewed along the a axis. Dashed lines indicate hydrogen bonds. H atoms not involed in hydrogen bonds have been omitted. Only the major disorder component is shown.

## supplementary crystallographic information

### Comment

Spiro-compounds are a particular class of naturally occurring substances characterized by highly pronounced biological properties (Kobayashi *et al.*, 1991; James *et al.*, 1991). The spiro-pyrrolidine ring system is also found in phermones, antibiotics (Gore *et al.*, 1991) and antitumour agents (Tietze *et al.*, 1988; Araki *et al.*, 2002). Indole compounds can be used as bioactive drugs (Stevenson *et al.*, 2000). Indole derivatives exhibit anti-allergic, central nervous system depressant and muscle relaxant properties (Harris & Uhle, 1960; Ho *et al.*, 1986). In view of this biological importance, the crystal structure of the title compound has been determined and the results are presented here.

A displacement ellipsoid plot of the title compound is shown in Fig. 1. The pyrrolizine ring system is folded about the bridging N1—C1 bond, as observed in related structures (Ramesh *et al.*, 2007). The sum of angles at N1 (339.7°) is in accordance with *sp*^3^ hybridization. The indole ring system (N2/C5/C14–C20) forms dihedral angles of 57.4 (6)° and 33.4 (5)°, respectively, with the C24—C29 and C8—C13 phenyl rings. The dihedral angle between the two phenyl rings is 82.9 (7)°. In the pyrrolizine ring system, the pyrrolidine ring (N1/C1/C5/C6/C7) adopts a twist conformation with Cremer & Pople (1975) puckering parameters q_2_ and φ of 0.419 (1) Å and 120.7 (2)°, respectively. Both major and minor conformers of the disordered pyrrolidine ring adopt envelope conformations; the puckering parameters q_2_ and φ are 0.267 (4) Å and -68.4 (8)°, respectively, for the major conformer (N1/C1-C4), and 0.254 (8) Å and 108.3 (8)°, respectively, for the minor conformer (N1/C1/C2/C3A/C4). Atom C3/C3A deviates by 0.411 (2)/0.389 Å from the N1/C1/C2/C4 plane.

The crystal structure is stabilized by intermolecular N—H···O hydrogen bonds and C—H···π interactions involving the C8-C13 phenyl ring (Table 1). The N—H···O hydrogen bonds link the molecules into centrosymmetric dimers (Fig. 2).

### Experimental

A solution of (1*E*,6*E*)-4-benzylidene-1,7-diphenylhepta-1,6-diene-3,5-dione (1 mmol), isatin (1 mmol) and *L*-proline (1 mmol) in aqueous methanol (20 ml) was refluxed until the disappearance of starting materials as evidenced by TLC. The solvent was removed under reduced pressure and the crude product was purified by column chromatography using petroleum ether-ethyl acetate (5:1) as eluent. The final product was recrystallized from ethanol-chloroform (2:8 v/v) solution.

### Refinement

Atom C3 of one of the pyrrolidine rings is disordered over two positions (C3 and C3A) with site occupancies of 0.546 (12) and 0.454 (12). All H atoms were placed in idealized positions and allowed to ride on their parent atoms, with N-H = 0.86 Å, C-H = 0.93-0.98 Å and *U*_iso_(H) = 1.2U_eq_(C).

### Crystal data

**Table:**

C_29_H_26_N_2_O_2_	*Z* = 2
*M**_r_* = 434.52	*F*_000_ = 460
Triclinic, *P*1	*D*_x_ = 1.261 Mg m^−^^3^
Hall symbol: -P 1	Mo *K*α radiation λ = 0.71073 Å
*a* = 8.4210 (2) Å	Cell parameters from 9449 reflections
*b* = 11.8895 (3) Å	θ = 2.3–30.1º
*c* = 12.5121 (3) Å	µ = 0.08 mm^−^^1^
α = 95.662 (1)º	*T* = 293 (2) K
β = 105.071 (1)º	Prism, yellow
γ = 105.815 (1)º	0.30 × 0.20 × 0.16 mm
*V* = 1144.31 (5) Å^3^	

### Data collection

**Table:**

Bruker Kappa APEXII diffractometer	7422 independent reflections
Radiation source: fine-focus sealed tube	4682 reflections with *I* > 2σ(*I*)
Monochromator: graphite	*R*_int_ = 0.024
*T* = 293(2) K	θ_max_ = 31.3º
ω scans	θ_min_ = 1.7º
Absorption correction: multi-scan(Blessing, 1995)	*h* = −12→11
*T*_min_ = 0.977, *T*_max_ = 0.987	*k* = −17→17
30483 measured reflections	*l* = −18→18

### Refinement

**Table:**

Refinement on *F*^2^	Secondary atom site location: difference Fourier map
Least-squares matrix: full	Hydrogen site location: inferred from neighbouring sites
*R*[*F*^2^ > 2σ(*F*^2^)] = 0.058	H-atom parameters constrained
*wR*(*F*^2^) = 0.200	*w* = 1/[σ^2^(*F*_o_^2^) + (0.1054*P*)^2^ + 0.1777*P*] where *P* = (*F*_o_^2^ + 2*F*_c_^2^)/3
*S* = 1.04	(Δ/σ)_max_ = 0.001
7422 reflections	Δρ_max_ = 0.43 e Å^−^^3^
308 parameters	Δρ_min_ = −0.23 e Å^−^^3^
Primary atom site location: structure-invariant direct methods	Extinction correction: none

### Special details

**Table:**

Geometry. All e.s.d.'s (except the e.s.d. in the dihedral angle between two l.s. planes) are estimated using the full covariance matrix. The cell e.s.d.'s are taken into account individually in the estimation of e.s.d.'s in distances, angles and torsion angles; correlations between e.s.d.'s in cell parameters are only used when they are defined by crystal symmetry. An approximate (isotropic) treatment of cell e.s.d.'s is used for estimating e.s.d.'s involving l.s. planes.
Refinement. Refinement of *F*^2^ against ALL reflections. The weighted *R*-factor *wR* and goodness of fit *S* are based on *F*^2^, conventional *R*-factors *R* are based on *F*, with *F* set to zero for negative *F*^2^. The threshold expression of *F*^2^ > σ(*F*^2^) is used only for calculating *R*-factors(gt) *etc*. and is not relevant to the choice of reflections for refinement. *R*-factors based on *F*^2^ are statistically about twice as large as those based on *F*, and *R*- factors based on ALL data will be even larger.

### Fractional atomic coordinates and isotropic or equivalent isotropic displacement parameters (Å^2^)

**Table:**

	*x*	*y*	*z*	*U*_iso_*/*U*_eq_	Occ. (<1)
O1	1.13350 (16)	0.65165 (11)	0.20138 (10)	0.0592 (3)	
O2	0.94660 (16)	0.99242 (9)	0.34629 (9)	0.0487 (3)	
N1	0.69132 (15)	0.79708 (12)	0.16496 (10)	0.0446 (3)	
N2	0.89750 (18)	0.84222 (11)	0.44618 (10)	0.0450 (3)	
H2	0.9240	0.8844	0.5119	0.054*	
C1	0.71391 (18)	0.77545 (15)	0.05236 (11)	0.0428 (3)	
H1	0.7437	0.8511	0.0259	0.051*	
C2	0.5380 (2)	0.6955 (2)	−0.02380 (15)	0.0623 (5)	
H2A	0.4778	0.7412	−0.0695	0.075*	0.546 (12)
H2B	0.5516	0.6330	−0.0733	0.075*	0.546 (12)
H2C	0.5382	0.6148	−0.0391	0.075*	0.454 (12)
H2D	0.5062	0.7225	−0.0937	0.075*	0.454 (12)
C3	0.4432 (6)	0.6455 (5)	0.0502 (4)	0.0542 (13)	0.546 (12)
H3A	0.4600	0.5698	0.0630	0.065*	0.546 (12)
H3B	0.3205	0.6329	0.0175	0.065*	0.546 (12)
C3A	0.4171 (6)	0.7056 (11)	0.0445 (4)	0.067 (2)	0.454 (12)
H3C	0.3658	0.7674	0.0245	0.080*	0.454 (12)
H3D	0.3248	0.6310	0.0294	0.080*	0.454 (12)
C4	0.5126 (2)	0.7336 (2)	0.15888 (16)	0.0635 (5)	
H4A	0.4454	0.7882	0.1586	0.076*	0.546 (12)
H4B	0.5095	0.6932	0.2224	0.076*	0.546 (12)
H4C	0.4673	0.7826	0.2006	0.076*	0.454 (12)
H4D	0.5076	0.6622	0.1895	0.076*	0.454 (12)
C5	0.83562 (17)	0.78291 (12)	0.25117 (11)	0.0367 (3)	
C6	0.97540 (17)	0.78955 (12)	0.18877 (10)	0.0342 (3)	
H6	1.0284	0.8732	0.1863	0.041*	
C7	0.86634 (17)	0.72579 (13)	0.06945 (10)	0.0364 (3)	
H7	0.8232	0.6407	0.0705	0.044*	
C8	0.95589 (18)	0.74035 (13)	−0.02018 (11)	0.0372 (3)	
C9	0.9407 (2)	0.64170 (14)	−0.09576 (12)	0.0463 (4)	
H9	0.8788	0.5664	−0.0889	0.056*	
C10	1.0168 (3)	0.65438 (17)	−0.18139 (14)	0.0571 (4)	
H10	1.0055	0.5875	−0.2315	0.069*	
C11	1.1081 (2)	0.76386 (18)	−0.19285 (14)	0.0575 (4)	
H11	1.1596	0.7715	−0.2502	0.069*	
C12	1.1240 (2)	0.86314 (17)	−0.11947 (14)	0.0547 (4)	
H12	1.1853	0.9381	−0.1275	0.066*	
C13	1.0487 (2)	0.85126 (14)	−0.03375 (12)	0.0459 (3)	
H13	1.0603	0.9187	0.0158	0.055*	
C14	0.89955 (18)	0.88773 (13)	0.35190 (11)	0.0381 (3)	
C15	0.8470 (2)	0.71800 (14)	0.42408 (12)	0.0434 (3)	
C16	0.8408 (3)	0.64255 (18)	0.50050 (15)	0.0600 (5)	
H16	0.8717	0.6717	0.5772	0.072*	
C17	0.7871 (3)	0.52212 (19)	0.45907 (19)	0.0714 (6)	
H17	0.7817	0.4691	0.5089	0.086*	
C18	0.7413 (3)	0.47874 (17)	0.34547 (18)	0.0669 (5)	
H18	0.7037	0.3971	0.3193	0.080*	
C19	0.7512 (2)	0.55614 (15)	0.27045 (15)	0.0539 (4)	
H19	0.7221	0.5268	0.1939	0.065*	
C20	0.80430 (19)	0.67704 (13)	0.30936 (12)	0.0410 (3)	
C21	1.11658 (18)	0.74122 (14)	0.24512 (12)	0.0415 (3)	
C22	1.2274 (2)	0.80951 (16)	0.35781 (13)	0.0486 (4)	
H22	1.2204	0.8845	0.3803	0.058*	
C23	1.3353 (2)	0.76867 (17)	0.42759 (14)	0.0516 (4)	
H23	1.3521	0.6986	0.4000	0.062*	
C24	1.4307 (2)	0.82413 (18)	0.54447 (14)	0.0542 (4)	
C25	1.3864 (3)	0.9102 (2)	0.60403 (15)	0.0654 (5)	
H25	1.2971	0.9376	0.5676	0.078*	
C26	1.4737 (3)	0.9552 (2)	0.71647 (17)	0.0810 (7)	
H26	1.4418	1.0114	0.7561	0.097*	
C27	1.6073 (3)	0.9165 (3)	0.7691 (2)	0.0931 (9)	
H27	1.6677	0.9477	0.8445	0.112*	
C28	1.6530 (3)	0.8331 (3)	0.7128 (3)	0.1039 (10)	
H28	1.7444	0.8078	0.7497	0.125*	
C29	1.5644 (3)	0.7853 (3)	0.6008 (2)	0.0830 (7)	
H29	1.5949	0.7268	0.5632	0.100*	

### Atomic displacement parameters (Å^2^)

**Table:**

	*U*^11^	*U*^22^	*U*^33^	*U*^12^	*U*^13^	*U*^23^
O1	0.0650 (8)	0.0620 (8)	0.0555 (7)	0.0293 (6)	0.0181 (6)	0.0059 (6)
O2	0.0677 (7)	0.0387 (6)	0.0321 (5)	0.0100 (5)	0.0121 (5)	−0.0012 (4)
N1	0.0375 (6)	0.0593 (8)	0.0301 (6)	0.0104 (5)	0.0082 (5)	−0.0051 (5)
N2	0.0613 (8)	0.0429 (7)	0.0250 (5)	0.0086 (6)	0.0145 (5)	−0.0029 (5)
C1	0.0404 (7)	0.0532 (9)	0.0283 (7)	0.0103 (6)	0.0067 (5)	−0.0011 (6)
C2	0.0431 (8)	0.0851 (14)	0.0402 (9)	0.0100 (8)	−0.0006 (7)	−0.0092 (9)
C3	0.0394 (18)	0.051 (2)	0.055 (2)	0.0010 (15)	0.0039 (14)	−0.0054 (17)
C3A	0.039 (2)	0.102 (6)	0.050 (2)	0.018 (2)	0.0014 (16)	0.011 (3)
C4	0.0388 (8)	0.0880 (14)	0.0545 (10)	0.0097 (8)	0.0153 (7)	−0.0037 (9)
C5	0.0395 (6)	0.0390 (7)	0.0254 (6)	0.0050 (5)	0.0105 (5)	−0.0041 (5)
C6	0.0375 (6)	0.0365 (7)	0.0237 (6)	0.0056 (5)	0.0092 (5)	−0.0005 (5)
C7	0.0411 (6)	0.0379 (7)	0.0237 (6)	0.0049 (5)	0.0091 (5)	−0.0015 (5)
C8	0.0425 (7)	0.0419 (8)	0.0240 (6)	0.0110 (6)	0.0090 (5)	0.0009 (5)
C9	0.0608 (9)	0.0423 (8)	0.0345 (7)	0.0121 (7)	0.0185 (7)	0.0004 (6)
C10	0.0781 (12)	0.0610 (11)	0.0378 (8)	0.0248 (9)	0.0267 (8)	−0.0002 (7)
C11	0.0687 (11)	0.0753 (12)	0.0351 (8)	0.0228 (9)	0.0258 (8)	0.0106 (8)
C12	0.0635 (10)	0.0562 (10)	0.0428 (9)	0.0092 (8)	0.0215 (8)	0.0129 (7)
C13	0.0559 (8)	0.0451 (8)	0.0334 (7)	0.0109 (7)	0.0154 (6)	0.0009 (6)
C14	0.0426 (7)	0.0405 (8)	0.0265 (6)	0.0086 (6)	0.0100 (5)	−0.0028 (5)
C15	0.0510 (8)	0.0436 (8)	0.0335 (7)	0.0086 (6)	0.0175 (6)	0.0022 (6)
C16	0.0809 (12)	0.0603 (11)	0.0423 (9)	0.0185 (9)	0.0265 (9)	0.0127 (8)
C17	0.1015 (16)	0.0559 (12)	0.0669 (13)	0.0207 (11)	0.0407 (12)	0.0247 (10)
C18	0.0915 (14)	0.0417 (10)	0.0708 (13)	0.0098 (9)	0.0420 (11)	0.0086 (9)
C19	0.0658 (10)	0.0420 (9)	0.0478 (9)	0.0030 (7)	0.0252 (8)	−0.0028 (7)
C20	0.0454 (7)	0.0407 (8)	0.0329 (7)	0.0050 (6)	0.0166 (6)	−0.0009 (6)
C21	0.0410 (7)	0.0495 (9)	0.0340 (7)	0.0113 (6)	0.0142 (6)	0.0075 (6)
C22	0.0454 (8)	0.0581 (10)	0.0400 (8)	0.0157 (7)	0.0098 (6)	0.0084 (7)
C23	0.0487 (8)	0.0655 (11)	0.0449 (9)	0.0204 (7)	0.0163 (7)	0.0158 (8)
C24	0.0418 (8)	0.0762 (12)	0.0399 (8)	0.0111 (7)	0.0089 (6)	0.0195 (8)
C25	0.0636 (11)	0.0808 (14)	0.0420 (9)	0.0191 (10)	0.0031 (8)	0.0104 (9)
C26	0.0912 (16)	0.0830 (16)	0.0447 (11)	0.0016 (12)	0.0090 (10)	0.0056 (10)
C27	0.0738 (14)	0.113 (2)	0.0494 (12)	−0.0149 (14)	−0.0117 (11)	0.0253 (13)
C28	0.0616 (13)	0.159 (3)	0.0767 (17)	0.0308 (16)	−0.0095 (12)	0.0473 (19)
C29	0.0599 (11)	0.121 (2)	0.0753 (15)	0.0402 (12)	0.0142 (11)	0.0323 (14)

### Geometric parameters (Å, °)

**Table:**

O1—C21	1.2092 (19)	C8—C13	1.389 (2)
O2—C14	1.2141 (18)	C9—C10	1.385 (2)
N1—C5	1.4610 (19)	C9—H9	0.93
N1—C4	1.467 (2)	C10—C11	1.362 (3)
N1—C1	1.4772 (18)	C10—H10	0.93
N2—C14	1.3477 (19)	C11—C12	1.375 (3)
N2—C15	1.398 (2)	C11—H11	0.93
N2—H2	0.86	C12—C13	1.381 (2)
C1—C2	1.525 (2)	C12—H12	0.93
C1—C7	1.529 (2)	C13—H13	0.93
C1—H1	0.98	C15—C16	1.375 (2)
C2—C3	1.443 (5)	C15—C20	1.387 (2)
C2—C3A	1.511 (6)	C16—C17	1.379 (3)
C2—H2A	0.97	C16—H16	0.93
C2—H2B	0.97	C17—C18	1.377 (3)
C2—H2C	0.96	C17—H17	0.93
C2—H2D	0.96	C18—C19	1.380 (3)
C3—C4	1.503 (4)	C18—H18	0.93
C3—H3A	0.97	C19—C20	1.379 (2)
C3—H3B	0.97	C19—H19	0.93
C3A—C4	1.402 (5)	C21—C22	1.481 (2)
C3A—H3C	0.97	C22—C23	1.318 (2)
C3A—H3D	0.97	C22—H22	0.93
C4—H4A	0.97	C23—C24	1.459 (2)
C4—H4B	0.97	C23—H23	0.93
C4—H4C	0.96	C24—C29	1.383 (3)
C4—H4D	0.96	C24—C25	1.394 (3)
C5—C20	1.511 (2)	C25—C26	1.380 (3)
C5—C14	1.5502 (18)	C25—H25	0.93
C5—C6	1.5623 (18)	C26—C27	1.367 (4)
C6—C21	1.502 (2)	C26—H26	0.93
C6—C7	1.5268 (18)	C27—C28	1.356 (4)
C6—H6	0.98	C27—H27	0.93
C7—C8	1.5044 (18)	C28—C29	1.382 (4)
C7—H7	0.98	C28—H28	0.93
C8—C9	1.387 (2)	C29—H29	0.93
			
C5—N1—C4	120.21 (14)	C8—C7—C6	116.26 (11)
C5—N1—C1	110.50 (11)	C8—C7—C1	114.40 (12)
C4—N1—C1	109.02 (12)	C6—C7—C1	101.23 (10)
C14—N2—C15	111.72 (11)	C8—C7—H7	108.2
C14—N2—H2	124.1	C6—C7—H7	108.2
C15—N2—H2	124.1	C1—C7—H7	108.2
N1—C1—C2	105.41 (12)	C9—C8—C13	117.87 (13)
N1—C1—C7	105.36 (11)	C9—C8—C7	119.99 (13)
C2—C1—C7	117.71 (14)	C13—C8—C7	122.08 (12)
N1—C1—H1	109.3	C10—C9—C8	120.57 (15)
C2—C1—H1	109.3	C10—C9—H9	119.7
C7—C1—H1	109.3	C8—C9—H9	119.7
C3—C2—C1	106.1 (2)	C11—C10—C9	120.64 (15)
C3A—C2—C1	103.4 (3)	C11—C10—H10	119.7
C3—C2—H2A	110.5	C9—C10—H10	119.7
C3A—C2—H2A	82.8	C10—C11—C12	119.90 (15)
C1—C2—H2A	110.5	C10—C11—H11	120.0
C3—C2—H2B	110.5	C12—C11—H11	120.0
C3A—C2—H2B	136.6	C11—C12—C13	119.82 (16)
C1—C2—H2B	110.5	C11—C12—H12	120.1
H2A—C2—H2B	108.7	C13—C12—H12	120.1
C3—C2—H2C	80.9	C12—C13—C8	121.20 (14)
C3A—C2—H2C	110.8	C12—C13—H13	119.4
C1—C2—H2C	111.0	C8—C13—H13	119.4
H2A—C2—H2C	131.3	O2—C14—N2	126.33 (12)
C3—C2—H2D	133.8	O2—C14—C5	125.50 (12)
C3A—C2—H2D	111.3	N2—C14—C5	108.16 (12)
C1—C2—H2D	111.2	C16—C15—C20	122.42 (15)
H2B—C2—H2D	80.9	C16—C15—N2	127.65 (14)
H2C—C2—H2D	109.1	C20—C15—N2	109.92 (13)
C2—C3—C4	106.6 (3)	C15—C16—C17	117.51 (17)
C2—C3—H3A	110.4	C15—C16—H16	121.2
C4—C3—H3A	110.4	C17—C16—H16	121.2
C2—C3—H3B	110.4	C18—C17—C16	121.39 (18)
C4—C3—H3B	110.4	C18—C17—H17	119.3
H3A—C3—H3B	108.6	C16—C17—H17	119.3
C4—C3A—C2	108.3 (3)	C17—C18—C19	120.12 (17)
C4—C3A—H3C	110.0	C17—C18—H18	119.9
C2—C3A—H3C	110.0	C19—C18—H18	119.9
C4—C3A—H3D	110.0	C20—C19—C18	119.81 (16)
C2—C3A—H3D	110.0	C20—C19—H19	120.1
H3C—C3A—H3D	108.4	C18—C19—H19	120.1
C3A—C4—N1	106.5 (3)	C19—C20—C15	118.74 (15)
N1—C4—C3	105.00 (19)	C19—C20—C5	132.59 (13)
C3A—C4—H4A	80.9	C15—C20—C5	108.62 (12)
N1—C4—H4A	110.7	O1—C21—C22	123.61 (15)
C3—C4—H4A	110.7	O1—C21—C6	121.49 (13)
C3A—C4—H4B	134.2	C22—C21—C6	114.88 (13)
N1—C4—H4B	110.7	C23—C22—C21	122.89 (16)
C3—C4—H4B	110.7	C23—C22—H22	118.6
H4A—C4—H4B	108.8	C21—C22—H22	118.6
C3A—C4—H4C	110.6	C22—C23—C24	125.45 (17)
N1—C4—H4C	110.6	C22—C23—H23	117.3
C3—C4—H4C	135.9	C24—C23—H23	117.3
H4B—C4—H4C	80.1	C29—C24—C25	118.35 (18)
C3A—C4—H4D	110.2	C29—C24—C23	118.88 (19)
N1—C4—H4D	110.3	C25—C24—C23	122.67 (16)
C3—C4—H4D	81.4	C26—C25—C24	120.8 (2)
H4A—C4—H4D	131.8	C26—C25—H25	119.6
H4C—C4—H4D	108.7	C24—C25—H25	119.6
N1—C5—C20	119.04 (12)	C27—C26—C25	119.4 (3)
N1—C5—C14	109.47 (12)	C27—C26—H26	120.3
C20—C5—C14	101.51 (11)	C25—C26—H26	120.3
N1—C5—C6	102.60 (10)	C28—C27—C26	120.9 (2)
C20—C5—C6	113.67 (12)	C28—C27—H27	119.6
C14—C5—C6	110.64 (11)	C26—C27—H27	119.6
C21—C6—C7	116.10 (12)	C27—C28—C29	120.4 (2)
C21—C6—C5	113.56 (11)	C27—C28—H28	119.8
C7—C6—C5	102.38 (10)	C29—C28—H28	119.8
C21—C6—H6	108.1	C28—C29—C24	120.2 (3)
C7—C6—H6	108.1	C28—C29—H29	119.9
C5—C6—H6	108.1	C24—C29—H29	119.9
			
C5—N1—C1—C2	134.55 (15)	C10—C11—C12—C13	0.6 (3)
C4—N1—C1—C2	0.4 (2)	C11—C12—C13—C8	−0.3 (3)
C5—N1—C1—C7	9.38 (16)	C9—C8—C13—C12	−0.2 (2)
C4—N1—C1—C7	−124.81 (15)	C7—C8—C13—C12	−177.30 (14)
N1—C1—C2—C3	−17.5 (4)	C15—N2—C14—O2	−175.92 (15)
C7—C1—C2—C3	99.5 (3)	C15—N2—C14—C5	2.62 (17)
N1—C1—C2—C3A	15.1 (5)	N1—C5—C14—O2	−55.88 (18)
C7—C1—C2—C3A	132.1 (5)	C20—C5—C14—O2	177.44 (14)
C3A—C2—C3—C4	−61.7 (4)	C6—C5—C14—O2	56.48 (19)
C1—C2—C3—C4	27.8 (5)	N1—C5—C14—N2	125.56 (13)
C3—C2—C3A—C4	72.5 (5)	C20—C5—C14—N2	−1.11 (15)
C1—C2—C3A—C4	−26.5 (8)	C6—C5—C14—N2	−122.08 (13)
C2—C3A—C4—N1	27.2 (8)	C14—N2—C15—C16	175.59 (17)
C2—C3A—C4—C3	−64.8 (6)	C14—N2—C15—C20	−3.20 (18)
C5—N1—C4—C3A	−146.1 (5)	C20—C15—C16—C17	−1.1 (3)
C1—N1—C4—C3A	−17.1 (6)	N2—C15—C16—C17	−179.71 (18)
C5—N1—C4—C3	−112.8 (3)	C15—C16—C17—C18	0.0 (3)
C1—N1—C4—C3	16.2 (4)	C16—C17—C18—C19	1.0 (4)
C2—C3—C4—C3A	69.8 (6)	C17—C18—C19—C20	−1.1 (3)
C2—C3—C4—N1	−27.4 (5)	C18—C19—C20—C15	0.1 (3)
C4—N1—C5—C20	18.66 (19)	C18—C19—C20—C5	176.93 (17)
C1—N1—C5—C20	−109.67 (14)	C16—C15—C20—C19	1.0 (2)
C4—N1—C5—C14	−97.34 (16)	N2—C15—C20—C19	179.89 (14)
C1—N1—C5—C14	134.34 (12)	C16—C15—C20—C5	−176.54 (15)
C4—N1—C5—C6	145.14 (14)	N2—C15—C20—C5	2.32 (17)
C1—N1—C5—C6	16.82 (15)	N1—C5—C20—C19	62.0 (2)
N1—C5—C6—C21	−162.45 (12)	C14—C5—C20—C19	−177.84 (17)
C20—C5—C6—C21	−32.59 (16)	C6—C5—C20—C19	−59.0 (2)
C14—C5—C6—C21	80.86 (15)	N1—C5—C20—C15	−120.88 (13)
N1—C5—C6—C7	−36.51 (13)	C14—C5—C20—C15	−0.74 (15)
C20—C5—C6—C7	93.36 (13)	C6—C5—C20—C15	118.07 (13)
C14—C5—C6—C7	−153.20 (12)	C7—C6—C21—O1	−5.4 (2)
C21—C6—C7—C8	−69.38 (16)	C5—C6—C21—O1	112.87 (15)
C5—C6—C7—C8	166.35 (12)	C7—C6—C21—C22	176.22 (12)
C21—C6—C7—C1	166.04 (12)	C5—C6—C21—C22	−65.48 (16)
C5—C6—C7—C1	41.76 (13)	O1—C21—C22—C23	−11.3 (3)
N1—C1—C7—C8	−157.82 (12)	C6—C21—C22—C23	167.04 (15)
C2—C1—C7—C8	85.07 (17)	C21—C22—C23—C24	−171.52 (15)
N1—C1—C7—C6	−31.99 (14)	C22—C23—C24—C29	−167.19 (19)
C2—C1—C7—C6	−149.10 (14)	C22—C23—C24—C25	16.6 (3)
C6—C7—C8—C9	132.52 (15)	C29—C24—C25—C26	−0.2 (3)
C1—C7—C8—C9	−109.94 (16)	C23—C24—C25—C26	176.07 (19)
C6—C7—C8—C13	−50.44 (19)	C24—C25—C26—C27	1.4 (3)
C1—C7—C8—C13	67.09 (18)	C25—C26—C27—C28	−1.2 (4)
C13—C8—C9—C10	0.3 (2)	C26—C27—C28—C29	−0.2 (4)
C7—C8—C9—C10	177.46 (15)	C27—C28—C29—C24	1.4 (4)
C8—C9—C10—C11	0.1 (3)	C25—C24—C29—C28	−1.2 (3)
C9—C10—C11—C12	−0.5 (3)	C23—C24—C29—C28	−177.6 (2)

### Hydrogen-bond geometry (Å, °)

**Table:**

*D*—H···*A*	*D*—H	H···*A*	*D*···*A*	*D*—H···*A*
N2—H2···O2^i^	0.86	2.02	2.854 (2)	162
C28—H28···Cg1^ii^	0.93	2.89	3.815 (3)	172

Symmetry codes: (i) −*x*+2, −*y*+2, −*z*+1; (ii) *x*−1, *y*, *z*−1.
